# Supplementary material for: Identification, Pathogenicity, and Genetic Diversity of Fusarium spp. Associated with Maize Sheath Rot in Heilongjiang Province, China
Source: Int J Mol Sci. 2022 Sep 16;23(18):10821. doi: 10.3390/ijms231810821 (PMC9501324; doi:10.3390/ijms231810821)
Supplement: Supplementary file 1 [file ijms-23-10821-s001.zip › ijms-1891802-supplementary.pdf]

Identification, Pathogenicity, and Genetic Diversity of *Fusarium* spp. Associated with Maize Sheath Rot in Heilongjiang Province, China

Xilang Yang[1], Xi Xu[1], Shuo Wang[1], Li Zhang[1], Guijin Shen[1], Haolin Teng[1], Chunbo Yang[1], Chunru Song[1], Wensheng Xiang[1,2], Xiangjing Wang[1,2*], Junwei Zhao[1*]

[1] Key Laboratory of Agricultural Microbiology of Heilongjiang Province, Northeast Agricultural University, No. 600 Changjiang Road, Xiangfang District, Harbin 150030, P.R. China

[2] State Key Laboratory for Biology of Plant Diseases and Insect Pests, Institute of Plant Protection, Chinese Academy of Agricultural Sciences, Beijing, P.R. China

Email: wangneau2013@163.com; guyan2080@126.com/zhaojunwei@neau.edu.cn

Contents:

Supplementary Table S1

Supplementary Table S2

Supplementary Table S3

Table S1. GenBank accession numbers of *Fusarium* isolates obtained from symptomatic maize leaf sheaths collected from Heilongjiang province and reference strains.

Isolates	Species	GenBank accession nos.	
		ITS	TEF-1α
SH-fjx51	*F. verticillioides*	OM955947	OM978746
SH-fqfa51	*F. verticillioides*	OM955948	OM978747
HA-jb53	*F. verticillioides*	OM955950	OM978748
QQ-jz22	*F. verticillioides*	OM955959	OM978749
QQ-qfy321	*F. verticillioides*	OM955961	OM978750
QQ-qfy322	*F. verticillioides*	OM955962	OM978751
QQ-qfy11	*F. verticillioides*	OM955963	OM978752
QQ-qfz41	*F. verticillioides*	OM955966	OM978753
SH-jx3111	*F. verticillioides*	OM955968	OM978754
SH-jx321	*F. verticillioides*	OM955969	OM978755
SH-jy11	*F. verticillioides*	OM955970	OM978756
SH-jy31	*F. verticillioides*	OM955971	OM978757
SH-jy321	*F. verticillioides*	OM955972	OM978758
SH-jz512	*F. verticillioides*	OM955973	OM978759
SH-ja3	*F. verticillioides*	OM955974	OM978760
SH-jx322	*F. verticillioides*	OM955975	OM978761
SH-jx33212	*F. verticillioides*	OM955976	OM978762
SH-jx33111	*F. verticillioides*	OM955977	OM978763
SH-jy3322	*F. verticillioides*	OM955978	OM978764
SH-jz513	*F. verticillioides*	OM955979	OM978765
SH-qf211	*F. verticillioides*	OM955980	OM978766
SH-qfz11	*F. verticillioides*	OM955981	OM978767
SH-qfz4	*F. verticillioides*	OM955982	OM978768
SH-qf2112	*F. verticillioides*	OM955983	OM978769
SH-qf212	*F. verticillioides*	OM955984	OM978770
SH-qfz5	*F. verticillioides*	OM955985	OM978771
SYS-jb4	*F. verticillioides*	OM955986	OM978772
SYS-qfy11	*F. verticillioides*	OM955991	OM978773
SYS-qfy51	*F. verticillioides*	OM955992	OM978774
SYS-qfx31	*F. verticillioides*	OM955995	OM978775
WC-qfb5	*F. verticillioides*	OM955996	OM978776
QQ-jb2	*F. verticillioides*	OM956018	OM978777
QQ-jx211	*F. verticillioides*	OM956022	OM978778
QQ-jx212	*F. verticillioides*	OM956023	OM978779
QQ-qfy112	*F. verticillioides*	OM956026	OM978780
QQ-qfy313	*F. verticillioides*	OM956028	OM978781
QTH-qfz11	*F. verticillioides*	OM956034	ON059707
QTH-qfz122	*F. verticillioides*	OM956035	OM978782
QTH-qfz132	*F. verticillioides*	OM956036	OM978783
SH-jx322	*F. verticillioides*	OM956038	OM978784

SH-jx33112	*F. verticillioides*	OM956039	OM978785
SH-jx3312	*F. verticillioides*	OM956040	OM978786
SH-jx333	*F. verticillioides*	OM956041	OM978787
SH-jx334	*F. verticillioides*	OM956042	OM978788
SH-yj31	*F. verticillioides*	OM956043	OM978789
SH-jy3323	*F. verticillioides*	OM956044	OM978790
SH-qf2	*F. verticillioides*	OM956046	OM978791
SH-qf21	*F. verticillioides*	OM956047	OM978792
SH-qf2111	*F. verticillioides*	OM956048	OM978793
SH-qf41	*F. verticillioides*	OM956049	OM978794
SH-qf422	*F. verticillioides*	OM956050	OM978795
SH-qfz3	*F. verticillioides*	OM956053	OM978796
SYS-j2	*F. verticillioides*	OM956054	OM978797
SYS-j211	*F. verticillioides*	OM956055	OM978798
SYS-jx21	*F. verticillioides*	OM956059	OM978799
SYS-jz111	*F. verticillioides*	OM956061	OM978800
SYS-qfy41	*F. verticillioides*	OM956068	OM978801
QQ-bjx222	*F. verticillioides*	OM956070	OM978802
SH-bjx31	*F. verticillioides*	OM956071	OM978803
SYS-bj22	*F. verticillioides*	OM956074	OM978804
SYS-bqfy51	*F. verticillioides*	OM956077	OM978805
SYS-lanjz111	*F. verticillioides*	OM956079	OM978806
QQ-lvqfy312	*F. verticillioides*	OM956080	OM978807
QTH-zqfz122	*F. verticillioides*	OM956081	OM978808
SH-zqf511	*F. verticillioides*	OM956082	OM978809
SH-zjx3311	*F. verticillioides*	OM956083	OM978810
SYS-qfx22	*F. tricinctum*	OM956065	OM978744
SYS-qfx222	*F. tricinctum*	OM956066	OM978745
QTH-qf11	*F. temperatum*	OM955967	OM978737
SYS-qfz1	*F. temperatum*	OM955993	OM978738
SYS-qfz52	*F. temperatum*	OM955994	OM978739
JMS-hb5	*F. temperatum*	OM956015	OM978740
QTH-qf13	*F. temperatum*	OM956032	OM978741
QTH-qfb3	*F. temperatum*	OM956033	OM978742
SYS-qfb53	*F. temperatum*	OM956064	OM978743
HA-j3	*F. subglutinans*	OM955949	OM978712
HA-jb512	*F. subglutinans*	OM955951	OM978713
HA-jx1	*F. subglutinans*	OM955953	OM978714
QQ-jx221	*F. subglutinans*	OM955954	OM978715
QQ-jx222	*F. subglutinans*	OM955955	OM978716
QQ-jy52	*F. subglutinans*	OM955956	OM978717
QQ-jz321	*F. subglutinans*	OM955957	OM978718
QQ-jz322	*F. subglutinans*	OM955958	OM978719
SYS-j312	*F. subglutinans*	OM955987	OM978720

SYS-j311	*F. subglutinans*	OM955988	OM978721
HA-j21	*F. subglutinans*	OM955997	OM978722
HA-j31	*F. subglutinans*	OM955998	OM978723
HA-j32	*F. subglutinans*	OM955999	OM978724
HA-j33	*F. subglutinans*	OM956000	OM978725
HA-j35	*F. subglutinans*	OM956001	OM978726
HA-j36	*F. subglutinans*	OM956002	OM978727
HA-j44	*F. subglutinans*	OM956008	OM978728
HA-jx312	*F. subglutinans*	OM956013	OM978729
QQ-ja1	*F. subglutinans*	OM956016	OM978730
QQ-ja2	*F. subglutinans*	OM956017	OM978731
QQ-jb23	*F. subglutinans*	OM956019	OM978732
QQ-jb51	*F. subglutinans*	OM956020	OM978733
QQ-jx223	*F. subglutinans*	OM956024	OM978734
SH-qfz22	*F. subglutinans*	OM956052	OM978735
SH-hjx3311	*F. subglutinans*	OM956078	OM978736
QQ-qfy332	*F. sporotrichioides*	OM955965	OM978709
QQ-qfz11	*F. sporotrichioides*	OM956030	OM978710
QQ-qfz112	*F. sporotrichioides*	OM956031	OM978711
HA-ja5	*F. solani*	OM956010	OM978706
HA-ja51	*F. solani*	OM956011	OM978707
SYS-qfz51	*F. solani*	OM956069	OM978708
SYS-qfx4	*F. proliferatum*	OM956067	OM978705
QQ-qfy33	FIESC	OM955964	OM978703
HA-jx311	FIESC	OM955952	OM978684
HA-j41	FIESC	OM956003	OM978685
HA-j411	FIESC	OM956004	OM978686
HA-j412	FIESC	OM956005	OM978687
HA-j42	FIESC	OM956006	OM978688
HA-j43	FIESC	OM956007	OM978689
HA-jx3	FIESC	OM956012	OM978690
HA-jx313	FIESC	OM956014	OM978691
QQ-jx21	FIESC	OM956021	OM978692
QQ-jz4	FIESC	OM956025	OM978693
QQ-qfy13	FIESC	OM956027	OM978694
QQ-qfy331	FIESC	OM956029	OM978695
SH-jy51	FIESC	OM956045	OM978696
SH-qfa1	FIESC	OM956051	OM978697
SYS-qfb51	FIESC	OM956063	OM978698
SH-bjx3321	FIESC	OM956072	OM978699
SH-bjx3322	FIESC	OM956073	OM978700
SYS-bjz12	FIESC	OM956076	OM978701
SH-jx24	*F. asiaticum*	OM956037	OM978682
SYS-jy31	*F. asiaticum*	OM956060	OM978702

SYS-jx222	*F. acuminatum*	OM955989	ON059703
SYS-jb511	*F. acuminatum*	OM956057	ON059704
SYS-jb521	*F. acuminatum*	OM956058	ON059705
SYS-bjx2221	*F. acuminatum*	OM956075	ON059706
**LS86**[a]	***F. asiaticum***	**MW423687**	**MW685833**
**NRRL 54218**	***F. acuminatum***	**HM068326**	**HM068316**
**NRRL 26419**	***F. equiseti***	**GQ505688**	**GQ505599**
**JS3**	***F. incarnatum***	**MT889972**	**MT895844**
**DHHJYK2**	***F. proliferatum***	**MN461565**	**MT265245**
**qadrul-fusaruim-tulip-1**	***F. solani***	**MN611433**	**MW995477**
**G1**	***F. sporotrichioides***	**MN160235**	**MN429078**
**PG 1-2**	***F. subglutinans***	**MT598164**	**MT598158**
**EFA313B**	***F. temperatum***	**KC179827**	**KC179825**
**RTN17**	***F. tricinctum***	**MK028863**	**MK032320**
**FPM03**	***F. verticillioides***	**MZ868200**	**MZ955277**
**IRAN 3512C**	***Alternaria junci-acuti***	**NR174907**	**MT187255**

[a]Bold accession numbers were generated in other studies.

Table S2. Geographic origins and number of *Fusarium* isolates recovered from symptomatic maize sheaths collected from 7 locations of Heilongjiang province in China as well as the number of *Fusarium* isolates used for pathogenicity test.

Number of *Fusarium* isolates[a]	Geographic origins							
	Shuangyashan city	Qiqihar city	Harbin city	Suihua city	Jiamusi city	Wuchang city	Qitaihe city	Total
F. verticillioides	12(4)	12(4)	1(1)	36(5)	0	1(1)	4(3)	66(18)
F. subglutinans	2(1)	10(2)	11(2)	2(1)	0	0	0	25(6)
FIESC	2(1)	5(1)	8(2)	4(1)	0	0	0	19(5)
F. temperatum	3(1)	0	0	0	1(1)	0	3(1)	7(3)
F. acuminatum	4(4)	0	0	0	0	0	0	4(4)
F. solani	1(1)	0	2(2)	0	0	0	0	3(3)
F. sporotrichioides	0	3(3)	0	0	0	0	0	3(3)
F. tricinctum	2(2)	0	0	0	0	0	0	2(2)
F. asiaticum	1(1)	0	0	1(1)	0	0	0	2(2)
F. proliferatum	1(1)	0	0	0	0	0	0	1(1)

[a]Numbers in parentheses are the number of corresponding *Fusarium* isolates used for pathogenicity test. The *Fusarium* species with a large number of isolates were selected based on location and haplotype, while those with a small number of isolates were all selected.

Table S3. *Fusarium* isolates used for pathogenicity test, and the diameter of lesion statistics of *Fusarium* isolates.

Species	Isolates	[a]Diameter of lesion
F. verticillioides	SYS-qfy11	0.5630
F. verticillioides	SYS-qfy51	0.5490
F. verticillioides	SYS-qfx31	0.7300
F. verticillioides	SYS-jz111	0.7050
F. verticillioides	QQ-jx211	0.6550
F. verticillioides	QQ-jb2	0.6750
F. verticillioides	QQ-jx212	0.4450
F. verticillioides	QQ-qfy313	0.8700
F. verticillioides	HA-jb53	0.8600
F. verticillioides	SH-zqf511	0.4900
F. verticillioides	SH-zjx3311	0.4150
F. verticillioides	SH-bjx31	0.4500
F. verticillioides	SH-qfz3	0.5750
F. verticillioides	SH-qf422	0.4000
F. verticillioides	WC-qfb5	0.3800
F. verticillioides	QTH-qfz11	0.5000
F. verticillioides	QTH-qfz122	0.6200
F. verticillioides	QTH-qfz132	0.4500
F. subglutinans	SYS-j312	0.4350
F. subglutinans	QQ-ja1	0.6700
F. subglutinans	QQ-ja2	0.5650
F. subglutinans	HA-j31	0.5350
F. subglutinans	HA-j32	0.5500
F. subglutinans	SH-qfz22	0.3700
FIESC	SYS-qfb51	0.3100
FIESC	QQ-qfy33	0.3800
FIESC	HA-jx311	0.4650
FIESC	HA-j41	0.3600
FIESC	SH-jy51	0.4180
F. temperatum	SYS-qfz1	0.4720
F. temperatum	JMS-hb5	0.6800
F. temperatum	QTH-qf11	0.5750
F. acuminatum	SYS-jx222	0.8150
F. acuminatum	SYS-jb511	0.8000
F. acuminatum	SYS-jb521	0.7950
F. acuminatum	SYS-bjx2221	0.490
F. solani	HA-ja5	0.6550
F. solani	HA-ja51	0.3247
F. solani	SYS-qfz51	0.5650
F. sporotrichioides	QQ-qfy332	0.7550
F. sporotrichioides	QQ-qfz11	0.5350

F. sporotrichioides	QQ-qfz112	0.5280
F. tricinctum	SYS-qfx22	0.9500
F. tricinctum	SYS-qfx222	0.9470
F. asiaticum	SH-jx24	0.6050
F. asiaticum	SYS-jy31	0.7830
F. proliferatum	SYS-qfx4	0.9400

[a]Diameter of lesion is the average value of 9 treatments.
